# Enhanced wound healing through hydrogel: *Arthrospira platensis *and *Chlorella vulgaris* in carboxymethyl cellulose/ carboxymethyl chitosan/gelatin matrix with decellularized amniotic membrane in male Wistar rats

**DOI:** 10.22038/ijbms.2025.88031.19013

**Published:** 2025

**Authors:** Rasoul Kheradmandi, Mohammad Kamalabadi Farahani, Morteza Alizadeh, Nariman Rezaei, Sepehr Zamani, Arian Ehterami, Majid Salehi

**Affiliations:** 1 Student Research Committee, School of Medicine, Shahroud University of Medical Sciences, Shahroud, Iran; 2 Regenerative Medicine Research Center, Shahroud University of Medical Sciences, Shahroud, Iran; 3 Department of Tissue Engineering, School of Medicine, Shahroud University of Medical Sciences, Shahroud, Iran; 4 Department of Tissue Engineering and Biomaterials, School of Advanced Medical Sciences and Technologies, Hamadan University of Medical Sciences, Hamadan, Iran; 5 Institute for Regenerative Medicine (IREM), University of Zurich, Zurich, Switzerland

**Keywords:** Tissue engineering, Wound healing, Skin regeneration, Scaffold, Arthrospira platensis, Chlorella vulgaris

## Abstract

**Objective(s):**

Wound healing requires effective biomaterials to overcome the limitations of conventional treatments, especially for full-thickness injuries. This study introduces an innovative hydrogel composed of carboxymethyl cellulose (CMC), carboxymethyl chitosan (CMCS), and gelatin (Gel), enhanced with extracts from* Arthrospira platensis* (AP) and *Chlorella vulgaris* (CV). The matrix is further integrated with decellularized amniotic membranes to enhance therapeutic effects.

**Materials and Methods:**

Hydrogels were formulated, crosslinked using 1-Ethyl-3-(3-Dimethylaminopropyl)carbodiimide (EDAC), and incorporated with 1% of either AP, CV, or both extracts. The scaffold was subjected to *in vitro* cell viability, red blood cell hemolysis, blood clotting index, and* in vivo* assays. The physical and chemical properties of the scaffolds were also evaluated using weight loss, swelling ratio, scanning electron microscopy (SEM), Fourier transform infrared (FTIR) spectroscopy, and nuclear magnetic Resonance (NMR) spectroscopy. To analyze wound healing under* in vivo *conditions, 36 male Wistar rats were used, and histopathological analysis was performed using hematoxylin and eosin staining.

**Results:**

*In vitro* studies demonstrated that AP-loaded hydrogels exhibited faster degradation and a higher release profile (85.25%) compared to CV (68.32%), consistent with AP’s anti-oxidant properties. I*n vivo* assessments on Wistar rats demonstrated that CV hydrogels achieved faster wound closure and better collagen synthesis, reaching 88 ± 2.5 % closure at 14 days versus 81 ± 2.64 % for AP (*P*<0.05). The CMC/CMCS/Gel/AP 1%/CV 1% hydrogels showed synergistic effects, achieving a 92 ± 2.1 % closure rate (*P*<0.01).

**Conclusion:**

The hydrogels demonstrated strong potential for skin repair, exhibiting good biocompatibility and controlled release; further refinement of the extracts and materials is suggested.

## Introduction

Skin wound healing is a complex biological process that requires interaction among several cell types to effectively repair skin tissue integrity. Cell migration, proliferation, and especially matrix deposition and remodeling are important stages in the successful healing of wounds (1). Unfortunately, because of their depth and length, certain skin wounds—which are frequently the consequence of trauma or serious injury—heal slowly, making them more prone to infection. As a result, within the past 20 years, there has been a noticeable increase in mortality linked to problems from insufficient skin tissue healing (2). Therefore, improving the wound-healing process has become a crucial focus of tissue engineering studies.

Polymeric hydrogels are complex three-dimensional structures that contain crosslinked networks rich in a vast amount of water, which is bound together through hydrogen-bonding interactions. Due to their exceptional hydrophilic properties and non-cytotoxicity toward cells, hydrogels made from polymers have proven to be valuable tools in tissue engineering (3). Hydrogel scaffolds composed of naturally derived macromolecules (4) or synthetic polymers (5) have great potential due to their biocompatible and biodegradable nature, as well as their inherent ability to interact with cells (6). 

Recent advances in the design and use of biodegradable hydrogels have led to significant progress in controlled drug delivery and tissue engineering. Due to their physicochemical properties, carboxymethyl cellulose (CMC)-based wound dressings are widely utilized. CMC is regarded as a chemical modification of cellulose and is one of the most significant polysaccharides found in nature. The hydroxyl groups of the glucopyranose chain of cellulose are joined by carboxymethyl groups (-CH_2_-COOH) in its chemical structure.

A well-known and biocompatible chitosan derivative, carboxymethyl chitosan (CMCS), possesses several beneficial qualities, including low toxicity, hydrophilicity, and biodegradability (7). Its uses include tissue engineering, medication delivery, and—most importantly—wound healing (8). Because of its rich arginine-glycine-aspartic acid (RGD) sequence, gelatin (Gel), another highly biocompatible polymer made from animal collagen, has a remarkable capacity to encourage cell adhesion and remodeling (9). Gelatin’s inferior mechanical qualities can be compensated for by mixing it with other polymeric materials, even though its characteristics are easily altered (10). Carboxymethyl chitosan can be added to tissue scaffolds to maximize its mechanical qualities (11).

*Arthrospira platensis *(AP) and *Chlorella vulgaris *(CV) are two types of algae that have received more attention in studies partly because of their potent anti-inflammatory and anti-oxidant properties. These green algae are commonly used in the food industry due to their high nutritional value, but researchers are also investigating potential applications in medicine. CV is rich in amino acids, unsaturated fatty acids, carbohydrates, carotenoid pigments, and chlorophyll (12). The composition of CV, however, may be influenced by several factors, including temperature, CO_2_, light exposure, and the type of algae being grown (13). Additionally, studies have shown that CV algae may possess anticancer properties (14). Despite its potential, the ability of CV algae to heal wounds has not yet been fully studied (15). On the other hand, it has been found that AP (Commonly known as *Spirulina*) modulates TGF-ß_1_, VEGF, bFGF, and TNF, which promotes angiogenesis and reduces scar formation (16). However, scientists suggest that a combination of these algae in hydrogels may enhance wound-healing outcomes (17). 

The amniotic membrane (AM), derived from the inner layer of the placenta, is a hard, elastic tissue that facilitates wound healing by serving as a supportive substrate rich in collagen and glycoprotein fibers, with a thin profile (0.02 to 0.05 mm) (18). The acellular amniotic membrane, for instance, is highly flexible and has been shown to facilitate wound healing by promoting an environment favorable to cell migration and proliferation (19). The rich extracellular matrix components of the acellular amniotic membrane have been reported to significantly improve wound closure, lower inflammation, and minimize scar formation (20).

Given the controllable mechanical properties, biocompatibility, and biodegradability of gelatin, carboxymethyl cellulose, and carboxymethyl chitosan, this study aims to examine the efficacy of their cost-effective combination, supplemented by extracts from CV and AP seaweed, for the repair of full-thickness wounds. It is suggested that an acellular amniotic membrane be added to the hydrogel to enhance its retention at the wound site for a longer period, thereby promoting and optimizing the wound-healing process.

## Materials and Methods

### Chemicals

The materials and solvents were bought from Merck (Darmstadt, Germany) and Sigma-Aldrich (St. Louis, USA), respectively. CMC (CAS no: 9004-32-4) and Gelatin powdered Type B from porcine skin (CAS no: 9000-70-8) were bought from Molekula. N-O carboxymethyl chitosan (90% acetylation degree) (Sahand Chemical Corp), AP, and CV powders were obtained from a certified local supplier (Tehran, Iran), each provided in vacuum-sealed, pharmaceutical-grade containers. According to the supplier’s certificate of analysis, both powders had a minimum purity of 90%, with a moisture content below 7%, and were free from microbial contamination.

### Extraction method of Arthrospira platensis and Chlorella vulgaris

According to de Melo *et al*. (21), for the isolation of CV, the following protocol was employed: after culturing in a mixotrophic condition, CV powder was attained and dissolved at a concentration of 50 mg/ml in 0.15 M Tris-HCl buffer (containing 25 mM NaCl, pH 7.5). The use of Tris-HCl buffer is warranted by the relatively strong and resistant cell wall of CV, for which a buffered state must be employed to provide a stable pH and ionic strength for the efficient recovery of proteins and peptides. The suspension was soaked for an hour and subsequently sonicated with 10 pulses at one-minute intervals employing a frequency of 20 kHz and 50% amplitude. The obtained solution was centrifuged for 10 min at 7000 rpm and 4 °C, and the supernatant was subsequently harvested and lyophilized for analysis. 

For AP, 3 g of mixotrophically grown powder was dissolved in 100 ml of deionized water and soaked for four hours. Unlike CV, the deionization of water as an extractant for AP fits perfectly due to its delicate cell membrane and heightened osmotic sensitivity, which minimizes the need for buffer reagents. This process not only safeguards the integrity of delicate bioactive compounds but also renders the extraction process cost-effective and easier. Sonication was finally carried out on the suspension using 5-second pulses followed by 15-second pauses for 30 min. The sample was then centrifuged for five minutes at 1500 rpm and 4 °C, and the supernatant was discarded. The pellet was lyophilized for use.

### Preparation of scaffolds

To prepare CMC/CMCs/Gel hydrogel, carboxymethyl cellulose (6% w/v) was dissolved in deionized water. Then, carboxymethyl chitosan (2% w/v) and gelatin (3% w/v) were dissolved separately in deionized water. Afterward, CMC, CMCs, and Gel were mixed in a ratio of 4:2:1 to achieve the appropriate hydrogel. After adding AP and CV to different samples of hydrogel, the samples were crosslinked with 1-ethyl-3-(3-dimethylaminopropyl) carbodiimide (EDAC) at a concentration of 0.01% w/v to crosslink the hydrogel matrix. The crosslinking of the CMC/CMCs/Gel hydrogel using EDAC involves a carbodiimide-mediated reaction that facilitates the formation of covalent amide bonds between carboxyl groups (–COOH) of carboxymethyl cellulose (CMC) and amino groups (–NH₂) present in chitosan and gelatin. This reaction results in the formation of a stable covalently crosslinked hydrogel network without introducing additional chemical residues into the matrix. Regarding biocompatibility, EDAC is considered a zero-length cross-linker, and based on previous studies, it minimizes potential cytotoxicity at the dosage used (22). Finally, the pH of the solutions was maintained at neutral during the crosslinking process using 1 M NaOH. For conducting specific *in vitro* tests, hydrogels were converted into scaffolds through the lyophilization process, which is described in detail for each test in advance.

### Scaffold characterization

*MTT assessment*

The MTT (The 3-(4, 5-dimethylthiazol-2-yl)-2,5-diphenyl-2H-tetrazolium bromide) test was done to determine cell viability and to figure out the optimum concentration of AP and CV extracts. Following the preparation of the hydrogels, they were individually supplemented with varying concentrations of AP and CV extracts to evaluate their effects on the hydrogel properties. For this purpose, hydrogels containing different AP concentrations were tested: 0.1% w/v (AP1), 1% w/v (AP2), 2.5% w/v (AP3), 5% w/v (AP4), and 10% w/v (AP5). Similarly, hydrogels containing different concentrations of CV were tested: 0.1% w/v (CV1), 1% w/v (CV2), 2.5% w/v (CV3), 5% w/v (CV4), and 10% w/v (CV5) For the MTT test, 3 ml of hydrogels with different AP and CV concentrations were mixed with 5 ml of cell culture medium for 24 hr. After that, 150 ul of their supernatant (liquid phase collected after incubating the hydrogels in culture medium for 24 hr, followed by allowing the system to settle to remove any residual solid or gel components) was collected and added to 7 × 10^3^ 3T3 cells already cultured into each 96-well plate for 24 and 72 hr. In this experiment, Dulbecco’s improved with Eagle’s medium: nutrient mixture F-12 was completed with 100 unit/ml of penicillin, 10% (v/v) FBS, and 100 µg/ml of streptomycin, and samples were kept in an incubator with 5% CO_2_ at 37 °C. In this test, the tissue culture plate was considered a positive control. To evaluate cell viability, MTT powder was dissolved in a cell culture medium (0.5 mg/mL). The culture medium of the samples was then substituted with 0.2 ml of MTT solution at 24 and 72 hr post-cell culturing. The plates were incubated in a dim location at 37 °C. After four hours, MTT solutions were substituted with DMSO to dissolve purple formazan crystals. For the final step, the absorption of samples was measured at 570 nm by a microplate reader (23).

The results of this study were used to select appropriate groups for subsequent characterization and studies. While dose-dependent effects have been observed, a concentration of 1% was selected for AP and CV due to the loss of hydrogel’s integrity properties at higher concentrations.

*Surface morphology analysis*

To determine the structure of CMC/CMCs/Gel, CMC/CMCs/Gel/AP 1%, CMC/CMCs/Gel/CV 1%, and CMC/CMCs/Gel/AP 1%/CV 1%, all hydrogel solutions were kept in the freezer at − 80 °C for 24 hr, and then to make a porous structure, all frozen solutions were freeze-dried (Telstar, Terrassa, Spain) at − 54 °C for 48 hr. After that, freeze-dried samples were cut (7 mm) and covered with gold by using a sputter coater (SCD 004, Balzers, Germany). A scanning electron microscope (SEM) (AIS2100, Seron Technology, South Korea) with an accelerating voltage of 15 kV was used to evaluate the structures of the samples. ImageJ (National Institutes of Health, Bethesda, USA) and Origin 22 software (OriginLab, Northampton, USA) were used to statistically calculate the average diameters of the pores. Analysis was conducted using a total of 20 random points per image. 

*Fourier transform infrared spectroscopy (FTIR) analysis*

FTIR analysis was conducted to assess the presence and stability of the chemical composition of the AP and CV during the hydrogel synthesis process. An FTIR spectrometer (WQF-510A, Rayleigh, China) was used to record IR spectra in the 400-4000 cm^-1^ range with a resolution of 4 cm^-1^.

*Nuclear magnetic resonance (NMR)*

^1^H nuclear magnetic resonance (^1^H NMR) spectra of hydrogels were recorded with a Bruker AvariceTM 500 NMR spectrometer to assess the degree of hydrophilic substitution of the polymer chains. For NMR measurement, the sample concentration was set as 1 mg/ml in distilled water.

*Evaluating in vitro degradation rate*

Freeze-dried hydrogels, CMC/CMCs/Gel, CMC/CMCs/Gel/AP 1%, CMC/CMCs/Gel/CV 1%, and CMC/CMCs/Gel/AP 1%/CV 1% were cut into 1 cm^2^ dimensions and weighed. Once their initial weights were recorded, they were kept in 10 cc of phosphate-buffered saline (PBS) (pH = 7.40) in an oven set at 37 °C for 2, 6, 24, and 48 hr. The aqueous solution was replaced at each time interval. After removal from the PBS, the specimens were incubated at 60 °C for a minimum of three days to dry. The final weights were recorded, and the degradation percentage of each specimen was calculated using the following equation (24).

$\mathrm{weight loss\&}=\frac{w_{0}-W_{1}}{W_{0}}\times 100$

In the above equation, W_0_ is the primary weight of hydrogels, and W_1_ is the dry weight after removal from the water. 

*Water absorption*

When determining the biodegradable materials to be used in drug delivery, it has been widely accepted that their swelling properties should be taken into consideration. The lyophilized hydrogel was quantified, and 5 mg of freeze-dried hydrogels were then added to 10 ml of PBS at room temperature for 2 days (specifically at 2, 6, 24, and 48 hr) at 37 °C. Samples were accessed systematically from the PBS and weighed immediately, with the weight being calculated (25).

*In vitro release of Arthrospira platensis and Chlorella vulgaris*

The release of AP and CV from samples was determined by UV–visible spectroscopy. The CMC/CMCs/Gel Hydrogels (1 ml) containing AP and CV were incubated into simulated body fluid (SBF) (5 ml) at 37 °C. During the defined period (2, 4, 6, 24, and 48 hr), approximately 1 ml of the supernatant was removed, and an equal volume of new SBF solution was added. The release content of AP and CV in the supernatant was determined by using a UV-VIS spectrophotometer at 450 nm and 680 nm to assess chlorophyll A and B, respectively. A standard curve methodology was employed using UV–visible spectroscopy. Calibration curves for chlorophyll a and chlorophyll b were constructed by preparing serial dilutions of known concentrations of each pigment standard. Absorbance measurements were taken at 450 nm for chlorophyll a and 680 nm for chlorophyll b, within the linear range of the spectrophotometer. The resulting calibration curves, typically linear with high correlation coefficients (R² > 0.99), were used to determine the concentrations of chlorophyll a and b in the hydrogel release samples by comparing their absorbance values at the respective wavelengths (26, 27).

*Assessment of pH changes*

Hydrogel solutions (CMC/CMCs/Gel, CMC/CMCs/Gel/AP 1%, CMC/CMCs/Gel/CV 1%, CMC/CMCs/Gel/AP 1%/CV 1%) were kept in the freezer at − 80 °C for 24 hr, and then, all frozen solutions were freeze-dried (Telstar, Terrassa, Spain) at − 54 °C for 48 hr. After that, freeze-dried samples were cut into 1 cm pieces. Freeze-dried samples with equal weight were submerged in PBS for 2, 4, 6, 24, 48, and 72 hr in order to perform pH assays. A pH meter 1140 from Mettler Toledo, Greisensee, Switzerland, was used to measure pH fluctuations at each interval.

*Hemolysis evaluation*

A technique adopted from Seyedi *et al*. was employed to assess the scaffolds’ hemolysis for scaffolds (CMC/CMCs/Gel, CMC/CMCs/Gel/AP 1%, CMC/CMCs/Gel/CV 1%, CMC/CMCs/Gel/AP 1%/CV 1%) (28). Two milliliters of fresh anticoagulated human blood and 2.5 ml of regular saline were combined. Following the addition of 0.2 ml of diluted blood to each sample, the specimens were promptly incubated for 60 min at 37 °C. The samples were then centrifuged at 1500 rpm for 10 min, and the supernatants were poured onto 96-well plates. A microplate reader (Synergy HTX Hybrid Multi-Mode Microplate Reader, BioTek, and Winooski, VT, USA) was used to assess each sample’s absorbance at 545 nm. 10 ml of normal saline was combined with 0.2 ml of diluted blood to create the negative control group. 10 ml of deionized water was mixed with 0.2 ml of diluted blood for the positive control group. The following formula was then used to determine the test samples’ hemolysis percentage (29).

$\mathrm{Hemolysis\&}=\frac{D_{t}-D_{\mathrm{nc}}}{D_{\mathrm{pc}}-D_{\mathrm{nc}}}\times 100$

The mean value of three measurements was reported, where Dt, Dnc, and Dpc represent the sample absorbance, negative control absorbance, and positive control absorbance, respectively.

*Blood coagulation index*

To calculate the blood coagulation index for the mentioned hydrogel samples, it is essential to prepare 2 ml of fresh human blood containing an anticoagulant. Following this, 100 mg of each sample is placed in a glass container for one hour in a water bath at 37 °C. Subsequently, 100 µl of blood containing anticoagulant is added to the samples, and after five minutes, 20 μl of a 0.2 mol/l CaCl_2_ solution is introduced. After another five minutes, 25 ml of distilled water is added to the samples, and then the samples are thoroughly mixed for five minutes at 37 °C. Finally, the absorbance of the samples is measured at 545 nm. The control group involves 100 μl of blood, along with 25 ml of distilled water (no sample). The following formula can be used to get the blood coagulation index:

$\mathrm{BCI}=\frac{A_{\mathrm{sample}}}{A_{\mathrm{control}}}\times 100$

In this formula, A_sample_ represents the absorption value of each sample, and A_control_ represents the absorption value of the control group (30).

*Total anti-oxidant capacity (TAC) measurement*

To measure the TAC for the mentioned hydrogel, samples were stored in the freezer at − 80 °C for 24 hr. To create a porous structure, all frozen solutions were then freeze-dried (Telstar, Terrassa, Spain) at − 54 °C for 48 hr. After that, the freeze-dried samples were cut, and a commercial kit from Zell Bio Company (Germany) was used, following the manufacturer’s protocol for the measurement of TAC (31). In this kit, ascorbic acid was utilized as the standard and benchmark for assessing the Total Anti-oxidant Capacity. Its sensitivity for measuring TAC is 0.1 mM. The TAC levels were calculated based on the standard curves established by the manufacturer’s instructions and expressed as micromoles per liter of tissue extract (µmol/l).

### Decellularization method of amnion membrane

The placenta used in this study was obtained from Bahar Hospital in Shahroud from mothers who gave birth via cesarean section (ethical code: IR.SHMU.AEC.1402.004). The placenta was then transferred to the university laboratory, where it was maintained in its integrity by placing it in a container containing 0.9% normal saline, along with 1% penicillin antibiotics, 1% streptomycin, and amphotericin B at a concentration of 2.5 mcg/ml, and surrounded by ice packs. Under aseptic conditions and a laminar hood, the placenta was washed three times with sterile PBS containing 1% penicillin, 1% streptomycin, and 2.5 μg/ml amphotericin B antibiotics to eliminate excess tissues, residual blood, and contamination. Subsequently, the amniotic membrane was separated from the chorionic membrane and other placenta parts and washed again with sterile PBS. The decellularization of the amniotic membrane was performed using the chemical-physical method, where the sample was immersed in 100 ml of EDTA 2% and incubated for 30 min at 37 °C. Then, the sample was immersed in 50 ml of 0.5 M NaOH for 30 sec, and a color change in tissue was observed. In the next step, the sample was immersed in 50 ml of 5% ammonium chloride for five minutes in a shaker. Finally, the acellular amnion membrane was placed on sterile foil and physically scraped on the surface with sterile gauze soaked in PBS. The acellular amnion membrane was stored at -20 °C for SEM imaging, DNA content analysis, and also DAPI staining (32).

*Investigating the morphology of amniotic membrane tissue*

The freeze-dried hydrogel samples and decellularized human amniotic membranes were sectioned into thin layers, each measuring 8 mm in diameter. These samples were subsequently sputter-coated with gold (Quorum Technologies, England) for 300 sec to enhance their imaging characteristics. The morphological evaluation of the samples was conducted using scanning electron microscopy (FE-SEM, MiRa3–TE scan, Czech Republic) at an accelerating voltage of 20 kV. To estimate the average pore size, 20 randomly selected points were analyzed per image using ImageJ software (National Institutes of Health, Bethesda, USA) in conjunction with OriginPro 2015 software (OriginLab, Northampton, USA).

*Examination of cell viability by DAPI staining*

To assess the presence of cell nuclei, DAPI staining was applied to both cellular and acellular amnion membranes. However, it should be emphasized that DAPI specifically binds to DNA and highlights nuclei and thus does not serve as a direct indicator of cell viability. Initially, a DAPI stock was prepared using H_2_O at a concentration of 10 mg/ml. Subsequently, the DAPI stock was diluted in PBS. At each sample site, 3 ml of sterile PBS solution was applied, followed by the addition of 10 μl of DAPI dye solution, which was then incubated at room temperature for 3 to 5 min. The samples were then aspirated and covered with a cover slip prior to observation, whereupon they were examined at λem ~461 nm.

*DNA content of acellular amniotic membrane*

To evaluate the cellular and DNA remnants from tissue samples, DNA extraction was performed utilizing the Pouya Gene Azma kit. The absorbance of DNA was quantified with a Biotek Cytation 5 plate reader/image reader at wavelengths of 260 and 280 nm (33).

### In vivo studies

**Figure 1:**
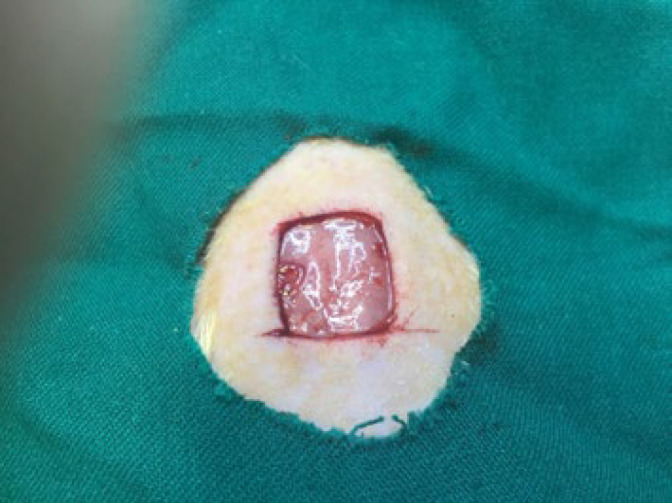
Visual documentation of full-thickness wound modeling in male Wistar rats

Shahroud University of Medical Science in Semnan, Iran, provided a total of 36 male Wistar rats in good health, each weighing roughly 250 g. CMC/CMCs/Gel, CMC/CMCs/Gel/AP 1%, CMC/CMCs/Gel/CV 1%, CMC/CMCs/Gel/AP 1%/CV 1%, negative control, and positive control were the six groups into which they were randomly assigned. The animal experiments were approved by the Ethics Committee of Shahroud University of Medical Sciences (IR.SHMU.AEC.1402.004) and were conducted following the university’s guidelines. The surgical process was conducted based on a previous study (34), which involved creating a full-thickness wound by making a 1.5 cm x 1.5 cm incision under general anesthesia conditions (Figure 1). Briefly, general anesthesia was induced by intraperitoneal injection of a mixture of Ketamine 5% and Xylazine 2% (70 mg ketamine and 6 mg xylazine/kg body weight). Hydrogels were applied directly to each rat using a sterile insulin syringe, and afterward, acellular amniotic membranes (cut to 1.5x1.5 cm^2^) were sutured on top of the hydrogels with 26 mm 3/8 circle reverse cutting non-absorbable nylon thread, considering high ethical standards. After 14 days of surgery, the animals were euthanized by intraperitoneal injection of 210 mg/kg ketamine hydrochloride and 18 mg/kg xylazine hydrochloride. The ImageJ software is used to calculate the wound closure area via equation (35):

$\mathrm{Wound clouser}\left(\%\right)=1-\frac{\mathrm{open wound area}}{\mathrm{initial wound area}}\times 100$

*Histological analysis*

Skin tissue samples were collected with utmost care and preserved by immersing them in 10% neutral buffered formalin (pH 7.2) for 48 hr. The fixed samples were then processed and embedded in paraffin before being sectioned to a thickness of 5 μm. These sections were further stained with hematoxylin and eosin (H&E) and were thoroughly evaluated under light microscopy (Olympus BX51; Olympus, Tokyo, Japan). The assessment of various parameters, including re-epithelialization and granulation tissue formation, was conducted in all groups to gain invaluable insights. Two independent, blinded dermatopathologists made a qualitative assessment of the stained sections to ensure objectivity and minimize observer bias. Blinding was ensured throughout the evaluation process to prevent any potential impact from knowledge of group allocation. The assessment targeted several important histopathological features that determine healing development, such as the integrity of the extracellular matrix (ECM), presence and formation of hair follicles and skin appendages, epidermal thickness, basement membrane development, vascularization of the dermal layer, and the rate of the overall wound closure. The specimens were examined using light microscopy by observers, and their observations were recorded against standardized descriptive criteria (36).

### Statistical analysis

The GraphPad Prism software (Version 10.4.1, Dotmatics, Boston, Massachusetts, USA) was used to perform the statistical analysis of the data. For group comparison, one-way analysis of variance (ANOVA) was conducted, and Tukey’s *post-hoc* test was applied. Data were compared using the Kruskal-Wallis H non-parametric ANOVA test, and the Mann-Whitney U test was applied in cases where significant differences were found. The mean ± standard deviation was used to represent the data. A *P*-value of less than 0.05 was considered statistically significant for every evaluation. 

## Results

### Cytotoxicity results

**Figure 2:**
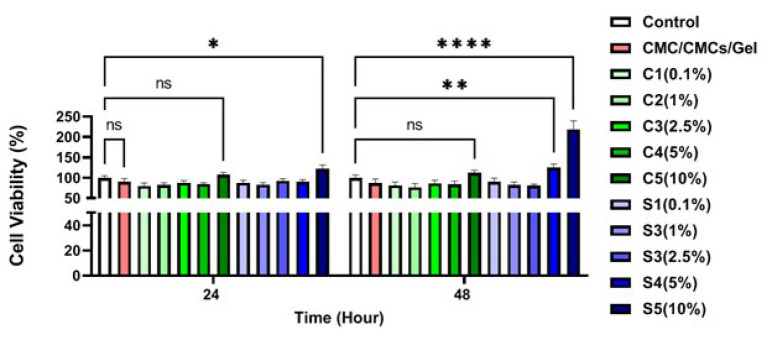
Cell viability histogram of mouse 3T3 fibroblast cells treated with CMC/CMCs/Gel hydrogel groups containing different concentrations of AP and CV at two incubation periods of 24 and 72 hr in male Wistar rats

An indirect MTT assay was used to evaluate the survival and proliferation of 3T3 murine fibroblastic cells in different scaffolds during 24 and 72 hr of cell culture. Figure 2 shows that the various amounts of CV and Sp extracts do not significantly differ from one another. However, compared to the control group, the concentrations of S4 (5%) at 48 hr and S5 (10%) showed a significant increase. The macroscopic structure of the CMC/CMCs/Gel hydrogel was shown to be disrupted by high concentrations of algae. Although direct rheological or mechanical analyses were not performed in this study, the observed changes in the structural integrity and displacement properties of CMC/CMCs/gel hydrogels at high concentrations strongly suggest disruption of their consistency and structure. To produce a hydrogel for *in vivo* research, a concentration of 1% was chosen for both AP and CV extracts. This choice was made in order to create a more stable hydrogel.

### Morphology evaluation

**Figure 3:**
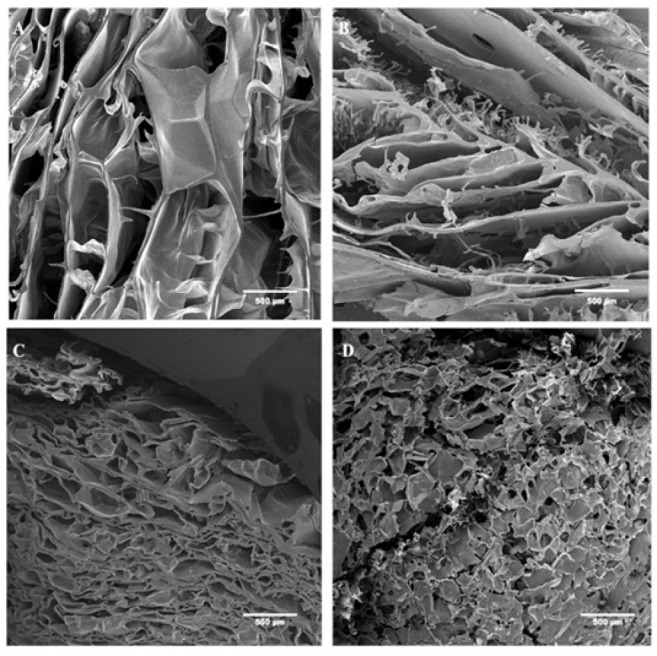
Scanning electron microscopy (SEM) images of the scaffold surfaces showing pore structure and size at 100× magnification: (A) CMC/CMCs/Gel, (B) CMC/CMCs/Gel with AP 1%, (C) CMC/CMCs/Gel with 1% CV and (D) CMC/CMCs/Gel with 1% AP and 1% CV

CMC: carboxymethyl cellulose; CMCs: carboxymethyl chitosan; Gel: gelatin; AP: *Arthrospira platensis*; CV: *Chlorella vulgaris*

The morphological and microstructural characteristics of CMC/CMCs/Gel hydrogel with different concentrations of AP and CV have been examined through SEM imaging, as shown in Figure 3. The results indicate that the hydrogel exhibits a highly porous structure. However, no interconnectivity was observed in AP and CV hydrogels, nor the group without algae. The average pore size of hydrogel and hydrogel containing AP 1%, CV 1%, and AP 1% + CV 1% was calculated 105.1 ± 45.6, 36.7 ± 14.2, 100 ± 25.4, and 56.7 ± 26.3 μm, respectively (Table1). 

### FTIR results

**Figure 4:**
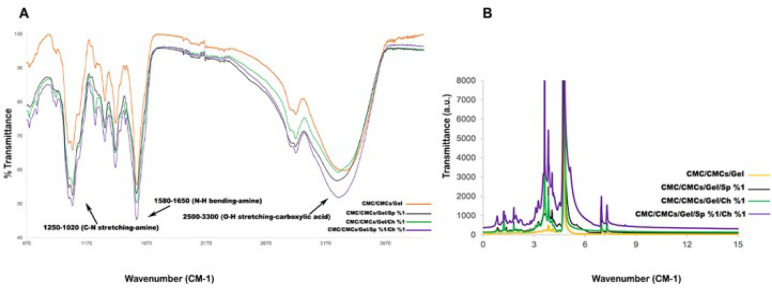
(A) Fourier-transform infrared (FTIR) spectroscopy spectra of various hydrogel formulations within the wavenumber range of 400–4000 cm⁻¹. (B) Proton nuclear magnetic resonance (¹H-NMR) spectra of the hydrogel samples

AP: *Arthrospira platensis*; CV: C*hlorella vulgari; *CMC: carboxymethyl cellulose; CMCs: carboxymethyl chitosan

The interaction of functional groups in CMC/CMCs/Gel, CMC/CMCs/Gel/AP 1%, CMC/CMCs/Gel/CV 1%, and CMC/CMCs/Gel/AP 1%/CV 1% was investigated by recording the FTIR spectrum in the range of 4000 to 400 cm^-1^ (Figure 4A). FTIR analysis identified the peaks at 3333 and 2918 which are related to amide groups, and carboxylic acid groups respectively. Furthermore, three additional peaks at 1412, 1323, and 1053 were observed, which corresponded to amine groups (37-39). The presence of these peaks indicates the structure of the CMC/CMCs/Gel hydrogel.

Spectroscopic analysis revealed the presence of additional various peaks in the range of 1496, 1244, 915, and 691, indicating C-H bending of the 1,2,4-trisubstituted and benzene derivative group, N-O stretching of the nitro compound group, and C-O stretching of the alkyl aryl ether group respectively (40-42). The presence of these peaks mentioned above suggests the potential involvement of AP and CV with CMC/CMCs/Gel hydrogel. Based on the FTIR findings, it can be concluded that both AP and CV extracts were properly loaded onto the hydrogel.

### H-NMR results

As depicted in Figure 4B, the CMC/CMCs/Gel hydrogel is formed by a combination of three polymers: carboxymethyl cellulose, carboxymethyl chitosan, and gelatin. To substantiate this assertion, the presence of the carboxymethyl group in the range of 4 to 4.5 ppm, the amide N-H bond in the range of 4.7 to 8.5 ppm, and the presence of aliphatic chains in the range of 0.8 to 3.5 ppm are required (43, 44). The introduction of AP has heightened the intensity of amide bond absorption because of the presence of aromatic protons from aromatic amino acids and pigments. Moreover, the intensity of carboxymethyl group absorption has increased in the hydrogel. Consequently, the drug release rate of AP was observed to be faster than that of CV. Furthermore, the active presence of AP and CV in the hydrogel is indicated by the presence of methyl and methylene protons in the range of 0.8 to 1.0 ppm and 1.2 to 1.6 ppm, respectively. In contrast to AP, it was shown that the hydrogel group containing CV had a reduced absorption intensity of the N-H amide bond. Finally, based on the domains shown in Figure 3, it can be said that both AP and CV have active sites for future connections and are optimally loaded onto the CMC/CMC/gel hydrogel. On the other hand, due to the presence of methyl and methylene groups, as well as the increased absorption intensity of the carboxymethyl group, the hydrogel group containing both AP and CV demonstrated a continuous and slower drug release rate compared to other groups.

### Weight loss

**Figure 5:**
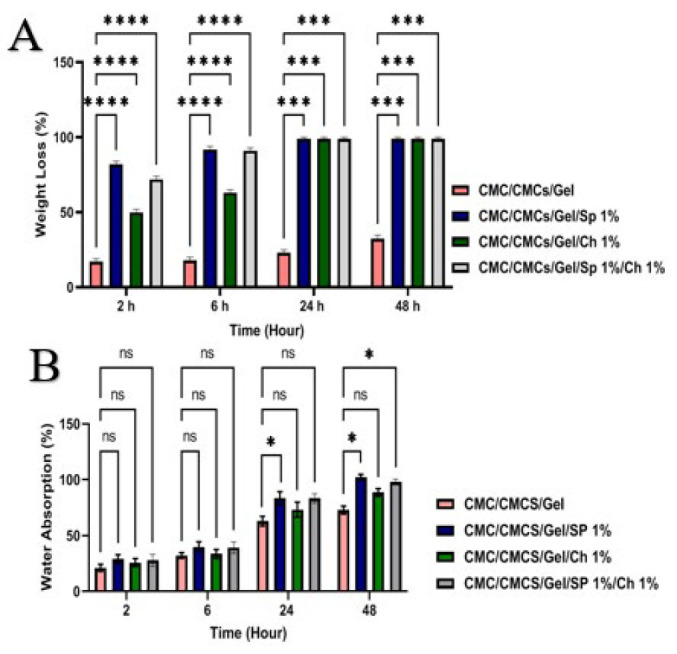
(A) Biodegradation of the scaffolds measured in phosphate-buffered saline (PBS) solution after 48 hr of incubation (%). (B) Water absorption capacity of the scaffolds measured after 48 hr of incubation Values represent the mean ± SD, n = 3. ns>0.05, *P*<0.05, ***P<0.001, ****P<0.0001

AP: *Arthrospira platensis*; CV: *Chlorella vulgaris*; SD: standard deviation; ns: not significant; p: probability value; CMC: carboxymethyl cellulose; CMCs: carboxymethyl chitosan

At 2, 6, 24, and 48-hr intervals, the weight loss percentages of the different scaffolds have been shown in Figure 5A. Over time, it was found that all scaffolds’ rates of degradation gradually rose. The addition of AP and CV to the CMC/CMCs/Gel hydrogel resulted in an accelerated rate of degradation, with 32% of the hydrogel degrading within 48 hr. However, after 24 hr, the hydrogels containing CV and AP were totally dissolved. Lastly, total degradation occurred in the group that included both AP and CV within a day. 

### Water absorption

Figure 5B shows the swelling behavior of CMC/CMCs/Gel hydrogels. The interaction between CMC/CMCs/Gel chains and water molecules can induce swelling of the polymer, which is limited by crosslinking. This swelling behavior of the CMC/CMCs/Gel hydrogel prepared a suitable 3D construct that mimics the native microenvironment of cells, thereby promoting cell survival, proliferation, and migration. AP extract is characterized by high levels of hydrophilic pigments and aromatic amino acids, including phycocyanin and phenols, which enhance the extract’s hydrophilicity. Increased water uptake is facilitated through enhanced swelling, and hydrolytic degradation of the polymer network is aided by improved mobility of the polymer chains and increased accessibility of water to hydrolyzable bonds. In contrast, CV has a more rigid cell wall structure composed of cellulose and glycoproteins, which results in reduced solubility and the ability to form stronger intermolecular interactions within the hydrogel matrix. Consequently, this leads to reduced water absorption and delayed swelling rate, observed as lower degradation and swelling values of the CV-comprising hydrogels. Additionally, the polar and aromatic components of AP can potentially reduce the overall crosslinking density by interacting with polymer chains and facilitating the initial loosening of the network, thereby accelerating hydrogel degradation further (45, 46). The results showed that the lowest and highest swelling rates of the hydrogels were observed in the CMC/CMCs/Gel and CMC/CMCs/Gel/AP 1% groups, with 72.99 ± 5.16% and 99.98 ± 4.73% swelling at 48 hr after incubation, respectively. 

### Release

**Figure 6:**
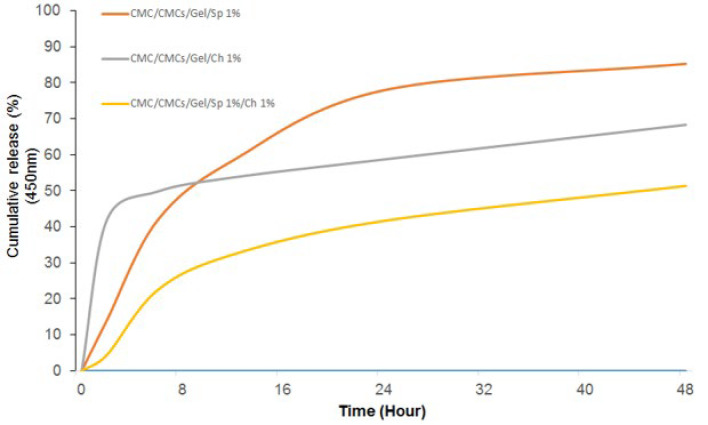
Cumulative release profile of AP and CV from hydrogel samples, measured in phosphate-buffered saline (PBS) solution at 450 nm over a period of 48 hr

Figure 6 illustrates the cumulative release properties of AP and CV. The hydrogel groups that contained AP, CV, and both AP+CV together exhibited the highest cumulative release rates, which were 85.25%, 68.32%, and 51.33%, respectively. In contrast to the other groups, the CV group’s burst release rate during the first two hours of release was significantly higher at 49.75%. On the other hand, the CV-containing hydrogel demonstrated a lower slope and a slower release rate. In contrast to CV, the AP group showed a slower release and a more durable drug release. Within 48 hr, the combination of both AP and CV produced the lowest slow release.

### pH results

**Figure 7:**
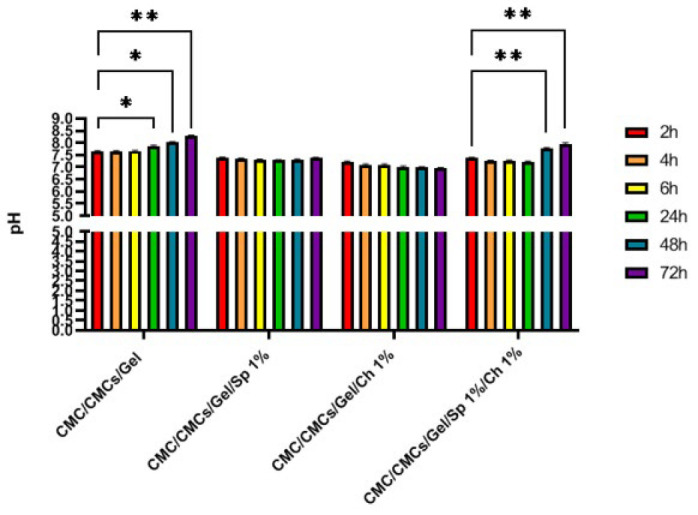
pH analysis of hydrogel samples after 72 hr of incubation

The pH variations in the PBS solution in contact with the scaffold were assessed at 2, 4, 6, 24, 48, and 72-hr intervals. The findings are illustrated in Figure 7. Overall, no statistically significant variances were observed among the hydrogel groups. However, the combined group displayed a notable contrast between the group between 48 and 72 hr as compared to the initial 2-hr timeframe (*P*<0.01). Conversely, the control group (CMC/CMCs/Gel hydrogel) exhibited an increase in alkalinity, with significant differences noted within the group at 24, 48, and 72-hr time points (*P*<0.01). It is worth noting that by adding AP and CV to the hydrogel, the pH of the hydrogel decreases slightly. It is worth noting that adding AP and CV to the hydrogel results in a non-significant decrease in pH.

### Hemocompatibility results

**Figure 8:**
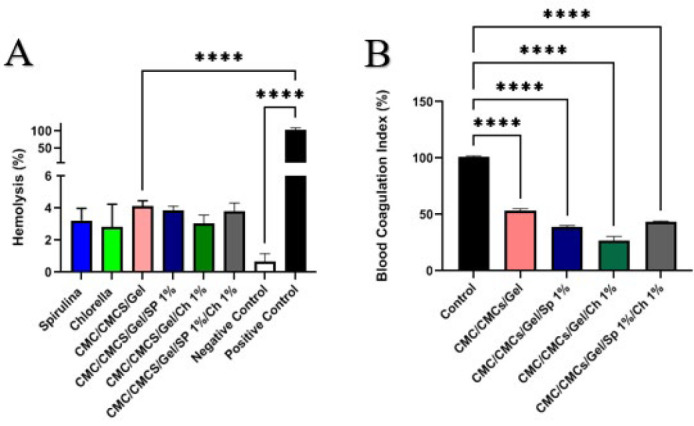
(A) Percentage of hemolysis caused by the prepared hydrogel samples (%). (B) Percentage of blood clotting index induced by the prepared hydrogels (%)

An important factor in influencing later responses to such inflammation is the interaction between the red blood cells and the wound dressing. Fresh red blood cells from a healthy person were used to test hydrogels containing AP and CV to assess their compatibility. A microplate reader was used to measure the amount of hemoglobin that was released. Direct contact with fresh human blood was used to evaluate the scaffolds’ blood compatibility; the resulting hemolysis percentage is displayed in Figure 8A. Statistical analysis revealed a significant disparity in the absorption rate between the positive control group and the scaffolds, indicating optimal compatibility of the scaffolds. All scaffolds exhibited a hemolysis percentage below 5%. The addition of any AP and CV into the bare hydrogel did not elicit a statistically significant positive or negative effect. Furthermore, no statistically significant variations were observed between the combined group and the bare hydrogel. 

### Blood coagulation index results

As shown in Figure 8B, the examination of the blood coagulation index test results indicates the existence of clot formation on the scaffolds. A decrease in the blood coagulation index suggests that the blood clotting process within the scaffolds is accelerated. Although this effect was less noticeable than that of the CMC/CMCs/Gel hydrogel group, Figure 8B shows that adding AP and CV to the scaffold improved platelet adherence and, as a result, encouraged clot formation. However, as the blood coagulation index value for this group was almost the same as that of the bare hydrogel group, the combination of AP and CV had no discernible effect on the index value.

### TAC results

**Figure 9:**
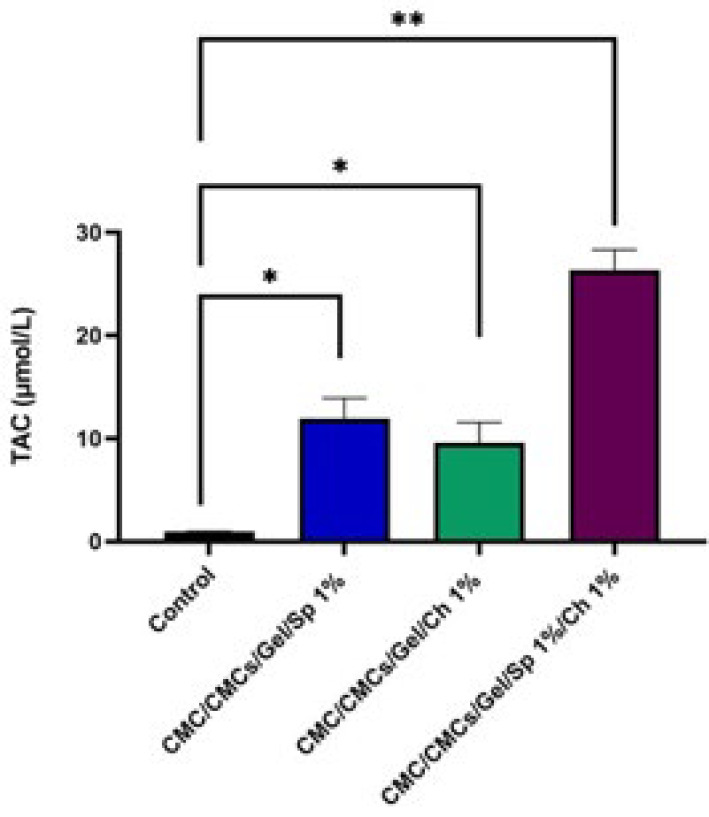
Total anti-oxidant capacity of the hydrogel samples

The total anti-oxidant capacity (TAC) for multiple samples is shown in Figure 9. The results show a significant increase in tissue extract TAC in the AP and CV-containing groups. The average TAC was 11.94 mol/l for the AP, 9.56 mol/l for the CV, and 26.33 mol/l for the combined samples. The findings indicate a significant difference from the control group. Additionally, there was a difference between the AP sample and the CV group, which was not significant (*P*<0.01). 

### Amniotic membrane SEM results

**Figure 10:**
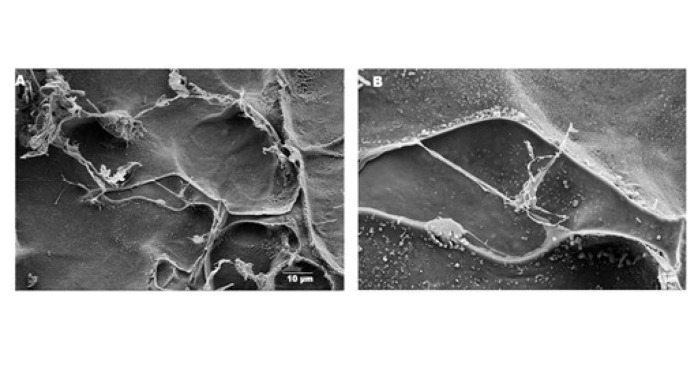
Scanning electron microscopy (SEM) images of the decellularized amniotic membrane at magnifications of (A) 2.5 K× and (B) 10 K×

The acellularization procedure was carefully performed under sterile conditions in compliance with the previously described procedures. The amniotic membrane’s SEM pictures are shown in Figure 10. The samples’ DNA content matches the data published in the Doudi *et al*. study (33), which revealed a significant decrease in the amount of residual DNA in acellular sample (<50 ng/mg of dry tissue) compared to cellular (1650 ng/mg respectively, *P*<0.05). The amniotic membrane tissue has been successfully decellularized, with its structure preserved, as indicated by the SEM image.

### DAPI staining of Amniotic membrane results

**Figure 11:**
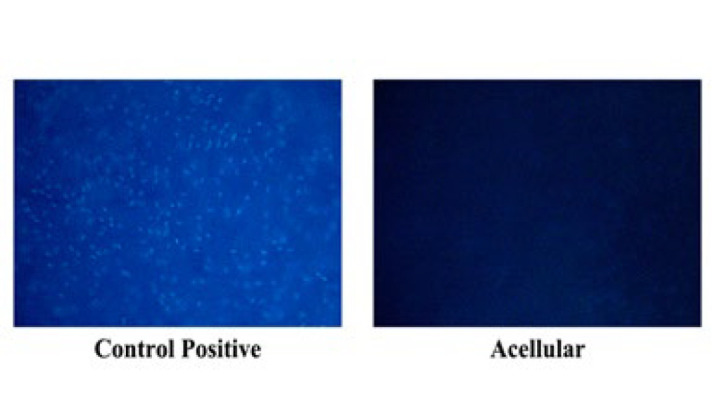
DAPI staining of the decellularized amniotic membrane observed at 100× magnification

Following the DAPI staining protocol, both decellularized and non-decellularized samples (control) are illustrated. According to the DAPI staining technique, the control sample is expected to exhibit numerous cells with a higher number of nuclei, while the decellularized sample should display fewer detectable nuclei. Figure 11 provides empirical evidence in support of this assertion, underscoring the efficacy of the decellularization process. 

### DNA content analysis

The SEM and DAPI assay results were confirmed by DNA quantitation, which also showed a remarkable reduction in the residual DNA content of the acellular amniotic membrane (<50 ng/mg dry tissue) concerning the non-acellularized and acellularized amniotic membranes (1572±102.88 ng/mg and 6.22±0.91 ng/mg, respectively; *P*<0.05) (47).

### In vivo wound healing study

**Figure 12:**
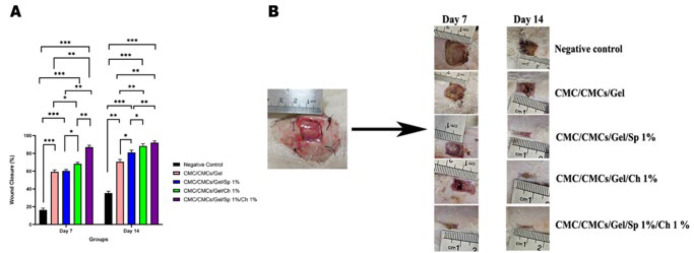
(A) Percentage of wound closure in different experimental groups of male Wistar rats at 7 and 14 days post-treatment. (B) Wound lesion area measured on the 7^th^ and 14^th^ days in different groups of male Wistar rats

As observed in Figure 12A, the CMC/CMCs/Gel hydrogel group significantly impacted wound closure, with 59.3 ± 2.08 % closure on the seventh day. AP and CV groups were able to significantly improve the wound healing process on the seventh day, with closure percentages of 60.3 ± 1.52% and 68.3 ± 1.6%, respectively. The combination group’s wound closure rate of 87.6 ± 2.0% on the seventh day was statistically significant when compared to the CMC/CMCs/Gel hydrogel group. However, a significant statistical difference was observed between AP and CV groups (*P*<0.01). On the 14^th^ day, the CMC/CMCs/Gel hydrogel group exhibited favorable wound healing performance, with an average closure of 70.6 ± 2.53%. AP and CV groups showed improvements, with average closure percentages of 81 ± 2.64% and 88 ± 2.5%, respectively. The combination group demonstrated an average closure of 92 ± 2.1 % on the 14^th^ day. On the 14^th^ day, however, a statistically significant difference was observed between the CV and AP groups (*P*<0.01). A macroscopic representation of the changes is shown in Figure 12B.

### Histopathological results

**Figure 13:**
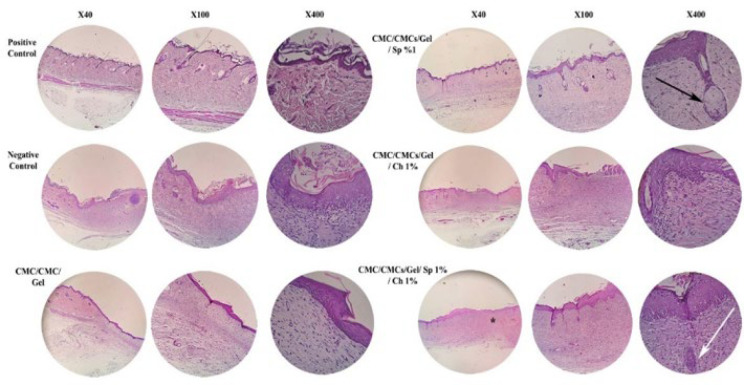
Microscopic sections of male Wistar rat skin tissue with healed incisions stained with H&E at 7 and 14 days post-treatment

The images labeled as Figure 13 show the H&E staining of lesion areas on day 14. The negative control group exhibits only loose connective tissue comprising collagen fibers, vessels, and fibroblasts. While this component is also present to varying degrees in other groups, the CMC/CMCs/Gel hydrogel group displays a more substantial and comparable extracellular matrix akin to the body’s natural skin tissue. Despite improvements of CMC/CMCs/Gel hydrogel compared to the negative control group, the groups containing AP and CV demonstrated a faster skin repair process than the CMC/CMCs/Gel hydrogel group. Notably, the AP hydrogel group features important elements such as hair follicles and skin appendages. The basement membrane is visible in both AP and CV-containing groups; however, the CV group exhibited a significantly thicker epidermal layer compared to the AP group. Furthermore, compared to the AP group, the CV group had a greater number of blood vessels in the dermis. Additionally, the combination group demonstrated the development of skin appendages, such as hair follicles, and the production of a basement membrane with the proper thickness, indicating that Sp and CV are clearly beneficial in healing skin tissue defects. Also, Table 2 presents qualitative microscopic observations of various histological parameters.

## Discussion

Rapid healing of full-thickness skin wounds remains a significant challenge in the field of medical science. Traditional approaches, such as surgical grafts, often fall short, especially when large areas of skin are damaged due to trauma, genetic disorders, or other causes. Consequently, tissue engineering—particularly through the use of hydrogels—has emerged as a promising strategy to address this problem. This study focuses on developing hydrogels incorporating AP and CV, two algae species known for their valuable biological properties, as agents to enhance skin regeneration.

Hydrogels are particularly attractive for tissue regeneration due to their intrinsic features, including high water retention, biocompatibility, biodegradability, and suitable porosity (48). Natural polymers, such as carboxymethyl cellulose, carboxymethyl chitosan, and gelatin, offer excellent compatibility with cells and controllable physical properties. Their biodegradability eliminates the need for manual removal from the wound site, making them ideal for skin repair applications (49). Both AP and CV have garnered attention in tissue engineering due to their anti-oxidant capabilities, affordability, and widespread availability. However, there has been a lack of comprehensive research on incorporating these algae into scaffolds specifically for skin wound healing. Their highly hydrophilic nature also poses challenges for stable hydrogel formulation. This study highlights the ability of AP- and CV-based hydrogels to create an optimal environment for cell migration, proliferation, and adhesion. The MTT assay results demonstrated that hydrogels containing these algae significantly improved the survival, adhesion, and proliferation of fibroblast cells at all tested time points. Prior studies have similarly demonstrated that the polymers used here have a positive effect on cell viability (50). AP extract, in particular, has been found non-toxic to skin fibroblasts while protecting against oxidative stress. Phycocyanin isolated from AP stimulates fibroblast proliferation and migration at concentrations below 150 μg/ml (51). Furthermore, *Spirulina* crude protein (SPCP) enhances the viability of human skin fibroblasts by activating the EGFR/MAPK pathways, promoting collagen synthesis, and inhibiting elastase activity (52). Nanofibers incorporating AP extract, such as alginate-PCL-AP composites, also demonstrate superior moisture retention and cell adhesion without toxicity to keratinocytes (53). Collectively, these findings indicate that AP algae extracts are safe and potentially beneficial for skin fibroblast function *in vitro*. Similarly, CV algae extracts have been extensively studied for their effects on skin fibroblasts. Extracts from CV and *Chlorella minutissima* exhibit anti-oxidant properties and reduce oxidative damage in human mesenchymal stem cells without impairing proliferation (54). Some species, such as *C. vulgaris* Roquette and Allma, exhibit negligible toxicity, whereas others, including C. sorokiniana variants, display varying fibrotoxicity (55). CV extracts also inhibit enzymes like elastase and tyrosinase, indicating their potential in combating skin aging and inflammation (56). Thus, CV extracts appear safe and valuable for skin care formulations. In this study, similar to AP, the highest fibroblast viability occurred at a 10% (w/v) concentration of CV, though this was not statistically different from controls. Concentrations of 5% and 10% AP in CMC/CMCs/Gel hydrogels showed significant positive effects; however, due to macroscopic structural issues at these concentrations, 1% AP and CV were chosen for *in vivo* experiments to maintain hydrogel integrity.

Fourier-transform infrared spectroscopy confirmed the hydrogel’s chemical composition and the successful incorporation of algae into the polymer scaffold. Shifts in peak positions indicated hydrogen bonding between algae compounds and hydrogel polymers, while the core chemical composition remained unchanged. Functional groups such as amide (-CONH₂-) and carboxymethyl (-COO-) identified in the hydrogels and algae are consistent with previous studies (57, 58). These groups enhance hydrophilicity and swelling capacity, which are crucial for drug delivery and wound healing (59). Stronger amide bonds in AP hydrogels suggest enhanced protein interactions and crosslinking, leading to controlled drug release and gradual hydrogel degradation (60).

The weight loss evaluation’s results indicate how fast hydrogels degrade in a physiological setting. The scaffold must degrade at an appropriate rate (61); if it degrades too quickly, the skin’s ability to maintain itself may be jeopardized before complete healing is possible. On the other hand, excessively slow degradation can prevent new cells from growing, thereby slowing down the healing process. The presence of AP increased hydrogel hydrophilicity and degradation rate compared to CV, which degrades more slowly and may better support longer tissue regeneration. Previous studies also report that AP enhances scaffold degradability (62, 63). Effective porous scaffolds typically have pore sizes ranging from 20 to 150 µm (64). AP reduced pore size more than CV, potentially affecting cell adhesion and migration—smaller pores may improve certain scaffold functions but hinder cell movement. 

Delivery of drugs relies substantially on cumulative release features. They enable a thorough assessment of drug delivery mechanisms by offering important insights into the manner and timing of drug release from bioscaffolds (65). Drug release profiles differed between the two algae: AP exhibited a slower initial release but achieved a higher cumulative release, whereas CV showed an early burst followed by sustained release. These findings align with those of Kim *et al*., who observed a ~20% release of AP-containing nanofibers within 4 hr (66). This suggests that AP hydrogels may be better suited for prolonged bioactive delivery, while CV is better suited for rapid drug release. AP’s superior hydrophilicity is likely due to its molecular composition, including higher aromatic amino acids and carboxymethyl groups, which aid water retention and maintain a moist wound environment—critical for faster healing and reduced infection risk. Recent research has demonstrated the tunable hydrophilicity of algae-containing hydrogels via carboxymethyl group modulation (67). Aliphatic side chain protons and -CH₂-COOH groups detected by NMR confirm algae purity in extractions (68). Our study showed that AP’s greater hydrophilicity correlates with more sustained drug release, beneficial for therapeutic applications, while CV also provided favorable early-release kinetics.

The pH test impacts a range of parameters, including biocompatibility, scaffold degradation, enzymatic activity, and drug delivery optimization. For hydrogel scaffolds used in skin regeneration, maintaining pH levels within a suitable range is vital to preserving the scaffolds’ structural integrity while providing an environment that is beneficial to skin tissue regeneration. (42). Maintaining a neutral to slightly alkaline pH in wounds is important for tissue regeneration since bacterial activity often increases acidity (69). AP and CV hydrogels showed no significant pH decrease, and their combination slightly increased alkalinity, potentially aiding in infection control and healing, especially in chronic wounds such as diabetic ulcers (70). Both algae possess potent anti-oxidant activity, vital for reducing oxidative stress at wound sites (71). AP exhibited higher total anti-oxidant capacity due to greater phenolic content. Combining AP and CV yielded the highest anti-oxidant activity, suggesting a synergistic effect in enhancing wound healing.

Blood compatibility is critical for scaffold safety, with hemolysis rates below 5% as the benchmark (72). AP and CV hydrogels demonstrated excellent blood compatibility and notable anticoagulant effects; AP notably inhibited fibrin formation, thereby reducing the risk of thrombosis (73). AP extract at various concentrations has shown anti-clotting effects (74), and both algae stimulate immune responses, such as phagocytosis and cytokine expression (75).

The decellularized amniotic membrane serves as an excellent scaffold in skin tissue engineering due to its high stem cell content, growth factors, and extracellular matrix, which support cell infiltration and tissue repair (76). Its low immunogenicity allows broad applications in burn and chronic wound healing (77). In this study, a physicochemical decellularization method was used to preserve the AM’s 3D structure, as confirmed by SEM and DNA quantification, which is consistent with previous protocols. The AM was sutured onto wounds on day one and removed after seven days following ethical guidelines. *In vivo*, CV-based hydrogels achieved superior wound closure (88%) compared to AP (81%), while their combination showed remarkable results. Interestingly, the CMC/CMCs/Gel hydrogel without additives also performed well, indicating its suitability for wound healing. While AP provides potent anti-oxidant and anti-inflammatory effects, it may be less effective for rapid closure, potentially due to protein degradation during crosslinking in gelatin nanofibers (78). Some studies reported no significant increase in collagen with AP ethanol extracts in diabetic rats (79), whereas nanophytosomal formulations of AP extracts enhanced healing more effectively (80).

One significant limitation of the current study is that it lacks a mechanical test, which is crucial in establishing the stability of the scaffold and tissue compatibility under physiological loading conditions. The mechanical characterization would have further implications for the load-bearing capability and durability over time of the scaffold in the body. Furthermore, while the study demonstrated anti-oxidant and anti-inflammatory effects, it lacked direct mechanistic investigations, including cytokine profiling and oxidative stress markers. To partially address this limitation, TAC measurements were performed, and the results were corroborated by an extensive review of the current literature. Future studies should incorporate these analyses to optimize scaffold performance and enhance wound healing in clinical applications.

A square, full-thickness wound (1.5x1.5 cm^2^) was created on the dorsal skin under general anesthesia, ensuring uniformity in depth and location

Values represent the mean ± SD, n = 6. ns > 0.05, ** *P*<0.01, ****P<0.0001. CMC: carboxymethyl cellulose; CMCs: carboxymethyl chitosan; Gel: gelatin; AP: *Arthrospira platensis; *CV: *Chlorella vulgaris*; SD: standard deviation; p: probability value

**Table 1:** Average pore size of hydrogels containing different concentrations of AP and CV. Values are presented as mean ± SD, n=3

Hydrogel	Average pore size (µm)
CMC/CMCs/Gel	105 ± 45.6
CMC/CMCs/Gel/Sp 1%	36.7 ± 14.2
CMC/CMCs/Gel/Ch 1%	100 ± 25.4
CMC/CMCs/Gel/Sp 1%/Ch 1%	56.7 ± 26.3

SD: standard deviation; AP: *Arthrospira platensis*; CV: *Chlorella vulgaris; *CMC: carboxymethyl cellulose; CMCs: carboxymethyl chitosan

nm: nanometer; AP: *Arthrospira platensis*; CV: *Chlorella vulgaris; *CMC: carboxymethyl cellulose; CMCs: carboxymethyl chitosan

Values are presented as mean ± SD, n = 3. AP: *Arthrospira platensis*; CV: *Chlorella vulgaris*; SD: standard deviation; CMC: carboxymethyl cellulose; CMCs: carboxymethyl chitosan

Values represent the mean ± SD, n = 3. **** *P*<0.0001. AP: *Arthrospira platensis*; CV: *Chlorella vulgaris*; SD: standard deviation; p: probability value; CMC: carboxymethyl cellulose; CMCs: carboxymethyl chitosan

Values represent the mean ± SD, n = 3. *P*<0.05, *P*<0.01. AP: *Arthrospira platensis*; CV: *Chlorella vulgaris*; SD: standard deviation; p: probability value; CMC: carboxymethyl cellulose; CMCs: carboxymethyl chitosan

DAPI: 4′,6-diamidino-2-phenylindole

Values represent the mean ± SD, n = 6. *P*<0.05, *P*<0.01, *P*<0.001. AP: *Arthrospira platensis*; CV: *Chlorella vulgaris*; SD: standard deviation; p: probability value; CMC: carboxymethyl cellulose; CMCs: carboxymethyl chitosan

Black arrow: sebaceous gland; White arrow: hair follicle; Star symbol: growing hair follicle. AP: *Arthrospira platensis*; CV: *Chlorella vulgaris*; H&E: hematoxylin and eosin staining; CMC: carboxymethyl cellulose; CMCs: carboxymethyl chitosan

**Table 2:** Qualitative assessment of key histopathological parameters involved in wound healing across different treatment groups of day 14 post-injury in male Wistar rats

Parameters	Negative control	CMC/CMCs/Gel	CMC/CMCs/Gel/SP 1%	CMC/CMCs/Gel/Ch 1%	CMC/CMCs/Gel/SP 1%/Ch 1%
Extracellular Matrix (ECM)	Loose connective tissue only	More substantial ECM, similar to natural skin tissue	Improved ECM	Improved ECM	Well-developed ECM
Hair follicles & appendages	Absent	Minimal	Present	Present	Present, well developed
Epidermal thickness	Thin	Moderate	Thinner than Ch group	Significantly thicker than previous groups	Proper thickness
Basement membrane	Not visible	Not clearly visible	Visible	Visible	Visible with proper thickness
Blood vessels in dermis	Few	Some	Less than Ch	More than Sp	Developed
Wound closure speed	Slow	Moderate	Faster than hydrogel alone	Faster than hydrogel alone	Fastest

AP: *Arthrospira platensis*; CV: *Chlorella vulgaris; *CMC: carboxymethyl cellulose; CMCs: carboxymethyl chitosan

## Conclusion

This study successfully developed and characterized bioactive hydrogels based on CMC/CMCs/Gel matrices incorporated with AP and CV extracts. Among all formulations, the hydrogels containing AP, CV, and AP+CV demonstrated enhanced bioactivity, including notable anti-oxidant capacity and sustained release profiles. Specifically, the AP-loaded hydrogels exhibited the highest cumulative release and biological performance, suggesting their potential for therapeutic applications. *In vitro* assessments confirmed that the hydrogels were structurally stable and blood-compatible, making them promising candidates for wound healing or transdermal drug delivery systems. Despite the absence of rheological and free extract hemolysis data, the results strongly suggest that embedding the bioactive agents in the hydrogel matrix enhanced safety and performance. In future studies, it is recommended to use each algae extract individually, in combination with hydrophobic polymeric scaffolds, to maintain bioactivity while minimizing the challenges associated with *in vitro* tests, such as release kinetics, hemolysis, and mechanical evaluation.

## Declaration

The authors wish to express their gratitude to the team behind DeepSeek, for their invaluable assistance in refining the language and enhancing the clarity of this manuscript. While the intellectual content and scientific contributions remain solely the responsibility of the authors, the support provided by DeepSeek is sincerely appreciated.
